# The Health of People with Intellectual Disability

**DOI:** 10.1155/2011/134571

**Published:** 2012-02-26

**Authors:** Nicholas Lennox, Henny Lantman, Robert Davis, Seeta Durvasula, Jacqueline Small, Margaret Kyrkou

**Affiliations:** ^1^Queensland Centre for Intellectual and Developmental Disability, School of Medicine, The University of Queensland, Brisbane, QLD 4072, Australia; ^2^Department of Primary and Community Care Centre for Family Medicine, Geriatric Care and Public Health, Radboud University Nijmegen Medical Centre, 6525 GA Nijmegen, The Netherlands; ^3^Centre for Developmental Disability Health, School of Primary Health Care, Victoria, Notting Hill, VIC 3168, Australia; ^4^Centre for Disability Studies, Northern Clinical School, Sydney Medical School, The University of Sydney, Camperdown, NSW 2050, Australia; ^5^Sydney Children's Hospital Network (Westmead), The University of Sydney, Westmead, NSW 2145, Australia; ^6^Department for Communities and Social Inclusion, Centre for Disability Health, Disability Services, Modbury, SA 5092, Australia

This special issue for the *International Journal of Family Medicine* is a first and warmly welcomed by those of us who work and research in the area.

In* “Prevalence of depression and dementia among adults with intellectual disabilities in Manitoba, Canada*,*”* Dr. S. Shooshtari and her coauthors have offered us a clear picture of both depression and dementia across the ages. Through data linkage across one Canadian province, Manitoba, the researchers examined specific data over five years about 6048 persons with developmental disability and then made comparisons with those of matched non-developmental disability. Younger adults with developmental disability were found to have a four-times higher risk of dementia than those without disability, and, in the older age group, the risk rises to more than five times. The pattern was similar for depression. Whilst the researchers acknowledge differences in diagnostic codes, they have provided us with a population-level picture of mental health conditions for people with developmental disability and a guide for future policy.

In* “Health and functional status of adults with intellectual disability referred to the specialist health care setting: a five-year experience*,” Dr. L. Lee and her coauthors examined the profile of 162 adults with intellectual disability who used a specialist outpatient clinic (Concord Hospital, Sydney) over five years. This extensive study found a great range of complexity of health needs, such as high prevalence of neurological dysfunction including epilepsy matched by low levels of physical activity, challenging behavior, and use of psychotropic medications. Older people with intellectual disability had higher rates of hospital stays with mental health issues and epilepsy management being the most common cause of hospitalizations. This paper provides the reader with a longitudinal examination of the complexity of health needs and healthcare servicing for people with intellectual disability. 

In “*The prevalence of depression among family caregivers of children with intellectual disability in a rural setting in Kenya*,” Dr. M. Njeri Mbugua and her coauthors report on their study of caregivers of children with intellectual disability and they found that nearly 80% of caregivers are at risk of clinical depression. Using the Beck Inventory and indicators of socioeconomic status, the researchers, supported by the Catholic Church, collected data from 114 caregivers in one parish in Kiambu (Kenya). Unemployment, low income, and family breakdown were indicators for higher risk, as were social isolation and stigma. 

In “*Parental perceptions of a Manchester Service for autistic spectrum disorders*,” Dr. M. Mockett and her coauthors present their findings from a survey of parents of children on the autism spectrum about their impressions of the multiagency Social Communication Assessment and Intervention Teams. Whilst most parents were happy with this assessment service, they placed emphasis on trust in their relationships with their children's health professionals. Communication between the teams, the parents and family, and the health professional was seen to be crucial.

In “*Parental concerns about the health of adolescents with intellectual disability: a brief report*,” Dr. M. Tucker and her coauthors have offered a brief insight into the health concerns that parents have for their adolescent child's future. The emerging themes from the qualitative arm of a cross-sectional descriptive study included concerns over dependency, general health, challenging behaviours, and increasing support needs. 

This first special issue on the health of people with intellectual disability reflects the breadth of research in the area and points policymakers to pitfalls in health service delivery.

*Nicholas Lennox* *Nicholas Lennox* *Henny Lantman* *Henny Lantman* *Robert Davis* *Robert Davis* *Seeta Durvasula* *Seeta Durvasula* *Jacqueline Small* *Jacqueline Small* *Margaret Kyrkou* *Margaret Kyrkou*

